# Aqua­{6,6′-dimeth­oxy-2,2′-[ethane-1,2-diylbis(nitrilo­methyl­idyne)]diphenolato}(4-hydroxy­benzoato)manganese(III)

**DOI:** 10.1107/S1600536809032553

**Published:** 2009-08-22

**Authors:** R. Reshma, P. V. Soumya, S. M. Simi, V. S. Thampidas, Robert D. Pike

**Affiliations:** a Department of Chemistry, SN College, Varkala, Kerala 695 145, India; b Department of Chemistry, College of William and Mary, PO Box 8795, Williamsburg, VA 23187-8795, USA

## Abstract

The title compound, [Mn(C_18_H_18_N_2_O_4_)(C_7_H_5_O_3_)(H_2_O)], was synthesized by a template reaction of ethane-1,2-diamine and 3-methoxy­salicylaldehyde in presence of manganese(II) 4-hydroxy­benzoate. The Jahn–Teller-distorted manganese(III) centre has an octa­hedral geometry. Extensive O—H⋯O hydrogen-bonding inter­actions generate a two-dimensional sheet structure parallel to (103).

## Related literature

For background to the coordination chemistry of manganese, see: Christou (2005 ▶); Yocum & Pecoraro (1999 ▶); McEvoy & Brudvig (2006 ▶); Pecoraro (1992 ▶). For the structures of manganese complexes containing Schiff base and carboxyl­ate ligands, see: Bermejo *et al.* (2006 ▶); Hulme *et al.* (1997 ▶); Zhang & Janiak (2001 ▶).
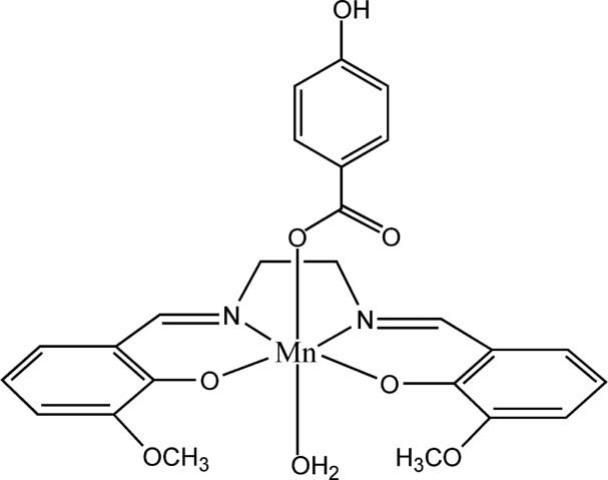


## Experimental

### 

#### Crystal data

[Mn(C_18_H_18_N_2_O_4_)(C_7_H_5_O_3_)(H_2_O)]
*M* *_r_* = 536.41
Monoclinic, 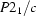

*a* = 8.5988 (3) Å
*b* = 13.5524 (5) Å
*c* = 21.1335 (8) Å
β = 93.280 (2)°
*V* = 2458.75 (16) Å^3^
*Z* = 4
Cu *K*α radiation
μ = 4.82 mm^−1^
*T* = 100 K
0.43 × 0.38 × 0.24 mm

#### Data collection

Bruker SMART APEXII CCD diffractometer
Absorption correction: numerical (*SADABS*; Sheldrick, 2004 ▶) *T*_min_ = 0.229, *T*_max_ = 0.388
26252 measured reflections
4259 independent reflections
3766 reflections with *I* > 2σ(*I*)
*R*_int_ = 0.033

#### Refinement

*R*[*F* ^2^ > 2σ(*F* ^2^)] = 0.029
*wR*(*F* ^2^) = 0.082
*S* = 1.04
4259 reflections
329 parameters
H-atom parameters constrained
Δρ_max_ = 0.17 e Å^−3^
Δρ_min_ = −0.31 e Å^−3^

### 

Data collection: *APEX2* (Bruker, 2004 ▶); cell refinement: *SAINT-Plus* (Bruker, 2004 ▶); data reduction: *SAINT-Plus*; program(s) used to solve structure: *SHELXS97* (Sheldrick, 2008 ▶); program(s) used to refine structure: *SHELXL97* (Sheldrick, 2008 ▶); molecular graphics: *ORTEP-3* (Farrugia, 1997 ▶) and *Mercury* (Macrae *et al.*, 2006 ▶); software used to prepare material for publication: *SHELXL97*.

## Supplementary Material

Crystal structure: contains datablocks global, I. DOI: 10.1107/S1600536809032553/bt5032sup1.cif

Structure factors: contains datablocks I. DOI: 10.1107/S1600536809032553/bt5032Isup2.hkl

Additional supplementary materials:  crystallographic information; 3D view; checkCIF report

## Figures and Tables

**Table 1:** Hydrogen-bond geometry (Å, °)

*D*—H⋯*A*	*D*—H	H⋯*A*	*D*⋯*A*	*D*—H⋯*A*
O8—H8⋯O5^i^	0.84	1.79	2.599 (2)	161
O3—H2*W*⋯O7^ii^	0.84	2.32	3.0025 (19)	139
O3—H2*W*⋯O2^ii^	0.84	2.11	2.8711 (17)	150
O3—H1*W*⋯O6^ii^	0.84	2.29	3.000 (2)	142
O3—H1*W*⋯O1^ii^	0.84	2.21	2.9475 (18)	147

Symmetry codes: (i) 
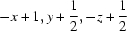
; (ii) 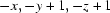.

**Fig. 1.**
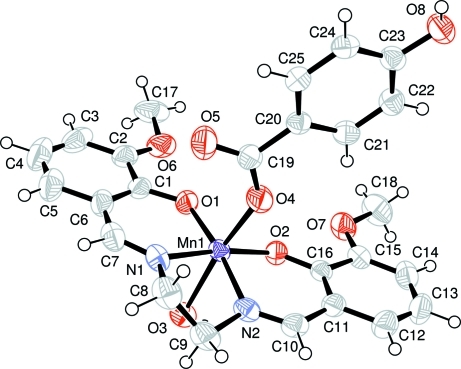
ORTEP picture (Farrugia, 1997) of the title compound. Displacement ellipsoids have been drawn at the 50% probability level.

**Fig. 2.**
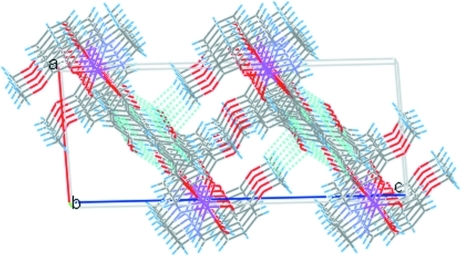
Mercury capped-stick packing diagram (Macrae et al., 2006) of the title compound showing hydrogen-bonding chains and the 2-dimensional sheet structure.

## supplementary crystallographic information

### Comment

Recent advances in the coordination chemistry of manganese have been mainly associated with (i) manganese clusters and the phenomenon of single-molecule magnetism (Christou, 2005) and (ii) its biological importance (Pecoraro, 1992). With its accessible oxidation states ranging from (II) to (V), and a propensity  for coordination with N and O donor atoms, manganese exhibits rich redox and structural chemistry in biological systems like the oxygen-evolvingcomplex (OEC) of photosystem II (McEvoy & Brudvig, 2006), superoxide dismutase, catalase, arginase etc. (Yocum & Pecoraro, 1999). We have been interested in inorganic modeling of the active sites of these manganese-containing systems using complexes containing Schiff base and carboxylate ligands. The structural diversity displayed in such complexes has been amply demonstrated in previous reports (Hulme *et al.*, 1997; Zhang & Janiak, 2001; Bermejo *et al.*, 2006). In this paper, we report the crystal structure of a new manganese(III)  complex with the Schiff base, m-salen [H_2_msalen = N,N'-bis(3-methoxysalicylidene)-ethane-1,2-diamine] and 4-hydroxyobenzoate as an ancillary ligand (Figure 1).

The N_2_O_2_ donor set of the m-salen ligand holds the manganese(III) ion at the centre of an approximate square plane [Mn(1)-O(1) = 1.8848 (12) Å and Mn(1)-O(2) = 1.8821 (11) Å ;Mn(1)-N(1) = 1.9774 (15) Å and Mn(1)-N(2) = 1.9930 (14) Å]. Jahn-Teller distortion elongate of the axial Mn–O_carb_ [Mn(1)-O(4) = 2.1164 (13) Å] and the Mn–O_aq_ [Mn(1)-O(3) = 2.3257 (12) Å]. H-bonding interactions between the non-coordinated O atom of the carboxylate and the para O-H group of the carboxylate of an adjacent molecule produce chains progressing along a screw (2_1_) axis parallel to the b-axis. Axial H_2_O ligands and the m-salen ligands of neighboring molecules are involved in multiple H-bond interactions resulting in chains. These two interactions together produce a 2-dimensional sheet structure parallel to the (1 0 3) plane (Figure 2).

### Experimental

To a solution of [Mn(4-OHC_6_H_4_CO_2_)(H_2_O)_2_].H_2_O (1.00 g, 2.61 mmol), and 3-methoxysalicylaldehyde (0.76 g, 5.22 mmol) in methanol (40 ml), ethane-1,2-diamine (0.16 g, 2.61 mmol) was added. The solution was stirred for 20 minutes, filtered and left to evaporation in an open conical flask. Brown crystals were deposited in 2–3 days. These were collected by filtration, washed with methanol, and dried in air. Crystals were grown from a DMF solution.

### Refinement

All hydrogen atoms were initially located in the difference map and then were placed in theoretical positions using a riding model. The methyl groups and the O-H groups were allowed to rotate but not to tip. C*sp*^2^—H = 0.95 Å, C*sp*^3^—H = 0.99 Å, *U*_iso_(H) = 1.2Ueq(C,O).

### Crystal data

**Table:**

[Mn(C_18_H_18_N_2_O_4_)(C_7_H_5_O_3_)(H_2_O)]	*F*(000) = 1112
*M**_r_* = 536.41	*D*_x_ = 1.449 Mg m^−^^3^
Monoclinic, *P*2_1_/*c*	Cu *K*α radiation, λ = 1.54178 Å
Hall symbol: -P 2ybc	Cell parameters from 305 reflections
*a* = 8.5988 (3) Å	θ = 9.7–72.7°
*b* = 13.5524 (5) Å	µ = 4.82 mm^−^^1^
*c* = 21.1335 (8) Å	*T* = 100 K
β = 93.280 (2)°	Block, red
*V* = 2458.75 (16) Å^3^	0.43 × 0.38 × 0.24 mm
*Z* = 4	

### Data collection

**Table:**

Bruker SMART APEXII CCD diffractometer	4259 independent reflections
Radiation source: fine-focus sealed tube	3766 reflections with *I* > 2σ(*I*)
graphite	*R*_int_ = 0.033
ω and ψ scans	θ_max_ = 67.0°, θ_min_ = 3.9°
Absorption correction: numerical (*SADABS*; Sheldrick, 2004)	*h* = −10→10
*T*_min_ = 0.229, *T*_max_ = 0.388	*k* = −16→16
26252 measured reflections	*l* = −25→23

### Refinement

**Table:**

Refinement on *F*^2^	Primary atom site location: structure-invariant direct methods
Least-squares matrix: full	Secondary atom site location: difference Fourier map
*R*[*F*^2^ > 2σ(*F*^2^)] = 0.029	Hydrogen site location: inferred from neighbouring sites
*wR*(*F*^2^) = 0.082	H-atom parameters constrained
*S* = 1.04	*w* = 1/[σ^2^(*F*_o_^2^) + (0.0482*P*)^2^ + 0.482*P*] where *P* = (*F*_o_^2^ + 2*F*_c_^2^)/3
4259 reflections	(Δ/σ)_max_ = 0.001
329 parameters	Δρ_max_ = 0.17 e Å^−^^3^
0 restraints	Δρ_min_ = −0.31 e Å^−^^3^

### Special details

**Table:**

Geometry. All e.s.d.'s (except the e.s.d. in the dihedral angle between two l.s. planes) are estimated using the full covariance matrix. The cell e.s.d.'s are taken into account individually in the estimation of e.s.d.'s in distances, angles and torsion angles; correlations between e.s.d.'s in cell parameters are only used when they are defined by crystal symmetry. An approximate (isotropic) treatment of cell e.s.d.'s is used for estimating e.s.d.'s involving l.s. planes.
Refinement. Refinement of *F*^2^ against ALL reflections. The weighted *R*-factor *wR* and goodness of fit *S* are based on *F*^2^, conventional *R*-factors *R* are based on *F*, with *F* set to zero for negative *F*^2^. The threshold expression of *F*^2^ > σ(*F*^2^) is used only for calculating *R*-factors(gt) *etc*. and is not relevant to the choice of reflections for refinement. *R*-factors based on *F*^2^ are statistically about twice as large as those based on *F*, and *R*- factors based on ALL data will be even larger.

### Fractional atomic coordinates and isotropic or equivalent isotropic displacement parameters (Å^2^)

**Table:**

	*x*	*y*	*z*	*U*_iso_*/*U*_eq_	
Mn1	0.00260 (3)	0.572439 (18)	0.395095 (12)	0.03435 (10)	
O1	0.16793 (14)	0.49020 (9)	0.42468 (6)	0.0441 (3)	
O2	0.00908 (13)	0.65071 (8)	0.46877 (5)	0.0385 (3)	
O3	−0.17820 (15)	0.46814 (10)	0.43826 (6)	0.0462 (3)	
H1W	−0.2082	0.4929	0.4719	0.069*	
H2W	−0.1324	0.4183	0.4539	0.069*	
O4	0.13396 (16)	0.68020 (10)	0.34910 (7)	0.0579 (4)	
O5	0.2768 (2)	0.61403 (11)	0.27687 (9)	0.0759 (5)	
O6	0.38532 (16)	0.39868 (11)	0.49052 (8)	0.0605 (4)	
O7	0.11387 (16)	0.74947 (10)	0.56553 (6)	0.0552 (4)	
O8	0.56714 (18)	1.04617 (10)	0.31546 (7)	0.0569 (4)	
H8	0.6043	1.0592	0.2805	0.085*	
N1	−0.03465 (18)	0.49221 (11)	0.31755 (7)	0.0437 (4)	
N2	−0.18769 (17)	0.64264 (11)	0.36080 (7)	0.0404 (3)	
C1	0.2130 (2)	0.40534 (13)	0.40135 (9)	0.0434 (4)	
C2	0.3325 (2)	0.35296 (14)	0.43583 (10)	0.0504 (5)	
C3	0.3854 (3)	0.26389 (16)	0.41349 (13)	0.0669 (7)	
H3A	0.4667	0.2299	0.4367	0.080*	
C4	0.3210 (3)	0.22341 (17)	0.35735 (13)	0.0716 (7)	
H4	0.3571	0.1617	0.3428	0.086*	
C5	0.2069 (3)	0.27213 (16)	0.32360 (12)	0.0648 (6)	
H5	0.1640	0.2442	0.2852	0.078*	
C6	0.1502 (2)	0.36359 (14)	0.34428 (9)	0.0486 (5)	
C7	0.0319 (3)	0.41113 (14)	0.30509 (9)	0.0503 (5)	
H7	0.0001	0.3795	0.2664	0.060*	
C8	−0.1493 (3)	0.53742 (16)	0.27184 (9)	0.0567 (5)	
H8A	−0.1983	0.4862	0.2439	0.068*	
H8B	−0.0976	0.5861	0.2451	0.068*	
C9	−0.2707 (2)	0.58766 (16)	0.30925 (9)	0.0528 (5)	
H9A	−0.3339	0.6332	0.2817	0.063*	
H9B	−0.3410	0.5381	0.3268	0.063*	
C10	−0.2353 (2)	0.72627 (14)	0.37993 (9)	0.0438 (4)	
H10	−0.3219	0.7552	0.3569	0.053*	
C11	−0.1684 (2)	0.77993 (13)	0.43343 (9)	0.0418 (4)	
C12	−0.2294 (3)	0.87431 (15)	0.44637 (11)	0.0568 (5)	
H12	−0.3083	0.9018	0.4184	0.068*	
C13	−0.1765 (3)	0.92618 (15)	0.49826 (12)	0.0620 (6)	
H13	−0.2186	0.9895	0.5063	0.074*	
C14	−0.0609 (3)	0.88696 (15)	0.53968 (10)	0.0554 (5)	
H14	−0.0246	0.9235	0.5759	0.067*	
C15	0.0010 (2)	0.79578 (13)	0.52835 (9)	0.0431 (4)	
C16	−0.05175 (19)	0.73910 (12)	0.47489 (8)	0.0363 (4)	
C17	0.5190 (2)	0.35790 (19)	0.52483 (14)	0.0759 (8)	
H17A	0.4945	0.2914	0.5395	0.114*	
H17B	0.5477	0.3999	0.5614	0.114*	
H17C	0.6063	0.3546	0.4970	0.114*	
C18	0.1804 (3)	0.8014 (2)	0.61902 (12)	0.0785 (7)	
H18A	0.2285	0.8625	0.6049	0.118*	
H18B	0.2598	0.7601	0.6411	0.118*	
H18C	0.0988	0.8173	0.6479	0.118*	
C19	0.2422 (2)	0.68274 (13)	0.31186 (9)	0.0416 (4)	
C20	0.33056 (19)	0.77892 (12)	0.31102 (8)	0.0363 (4)	
C21	0.3153 (2)	0.84743 (13)	0.35946 (8)	0.0418 (4)	
H21	0.2480	0.8334	0.3924	0.050*	
C22	0.3962 (2)	0.93532 (14)	0.36026 (9)	0.0443 (4)	
H22	0.3863	0.9805	0.3942	0.053*	
C23	0.4923 (2)	0.95794 (13)	0.31152 (9)	0.0404 (4)	
C24	0.5079 (2)	0.89109 (13)	0.26270 (8)	0.0408 (4)	
H24	0.5728	0.9061	0.2291	0.049*	
C25	0.4281 (2)	0.80217 (13)	0.26320 (8)	0.0392 (4)	
H25	0.4407	0.7561	0.2300	0.047*	

### Atomic displacement parameters (Å^2^)

**Table:**

	*U*^11^	*U*^22^	*U*^33^	*U*^12^	*U*^13^	*U*^23^
Mn1	0.03304 (16)	0.03382 (16)	0.03536 (16)	−0.00183 (11)	−0.00532 (11)	−0.00038 (11)
O1	0.0386 (7)	0.0387 (7)	0.0539 (7)	0.0051 (5)	−0.0062 (6)	−0.0046 (6)
O2	0.0397 (6)	0.0372 (6)	0.0374 (6)	0.0035 (5)	−0.0080 (5)	−0.0011 (5)
O3	0.0461 (7)	0.0452 (7)	0.0467 (7)	−0.0033 (6)	−0.0027 (6)	0.0103 (6)
O4	0.0593 (9)	0.0451 (7)	0.0719 (9)	−0.0112 (6)	0.0266 (7)	−0.0016 (7)
O5	0.0862 (12)	0.0505 (9)	0.0951 (12)	−0.0120 (8)	0.0399 (10)	−0.0203 (9)
O6	0.0405 (8)	0.0569 (8)	0.0830 (11)	0.0106 (6)	−0.0075 (7)	0.0137 (8)
O7	0.0569 (8)	0.0565 (8)	0.0497 (7)	−0.0026 (7)	−0.0175 (6)	−0.0100 (6)
O8	0.0603 (9)	0.0460 (7)	0.0651 (9)	−0.0143 (7)	0.0111 (7)	−0.0042 (7)
N1	0.0502 (9)	0.0412 (8)	0.0392 (8)	−0.0086 (7)	−0.0021 (7)	−0.0021 (7)
N2	0.0379 (8)	0.0449 (9)	0.0371 (7)	−0.0047 (6)	−0.0085 (6)	0.0066 (6)
C1	0.0378 (9)	0.0355 (9)	0.0581 (11)	0.0001 (7)	0.0132 (8)	0.0047 (8)
C2	0.0412 (10)	0.0435 (10)	0.0679 (13)	0.0014 (8)	0.0146 (9)	0.0104 (9)
C3	0.0585 (13)	0.0509 (12)	0.0944 (18)	0.0176 (10)	0.0311 (13)	0.0254 (13)
C4	0.0944 (18)	0.0417 (12)	0.0826 (17)	0.0110 (12)	0.0400 (15)	0.0024 (12)
C5	0.0864 (17)	0.0414 (11)	0.0694 (14)	−0.0010 (11)	0.0304 (13)	−0.0017 (10)
C6	0.0570 (12)	0.0366 (9)	0.0541 (11)	−0.0043 (8)	0.0186 (9)	−0.0033 (8)
C7	0.0621 (13)	0.0444 (11)	0.0449 (10)	−0.0118 (9)	0.0073 (9)	−0.0065 (8)
C8	0.0667 (14)	0.0609 (12)	0.0401 (10)	−0.0046 (11)	−0.0168 (10)	−0.0015 (9)
C9	0.0493 (12)	0.0608 (12)	0.0458 (10)	−0.0047 (9)	−0.0201 (9)	0.0019 (9)
C10	0.0368 (9)	0.0456 (10)	0.0481 (10)	0.0038 (8)	−0.0059 (8)	0.0119 (8)
C11	0.0382 (9)	0.0382 (9)	0.0487 (10)	0.0010 (7)	−0.0006 (8)	0.0060 (8)
C12	0.0538 (12)	0.0460 (11)	0.0704 (14)	0.0108 (9)	0.0013 (10)	0.0096 (10)
C13	0.0704 (15)	0.0392 (11)	0.0774 (15)	0.0079 (10)	0.0126 (12)	−0.0028 (10)
C14	0.0654 (13)	0.0431 (11)	0.0581 (12)	−0.0072 (10)	0.0063 (10)	−0.0081 (10)
C15	0.0423 (10)	0.0422 (10)	0.0448 (10)	−0.0070 (8)	0.0008 (8)	−0.0009 (8)
C16	0.0333 (8)	0.0353 (9)	0.0404 (9)	−0.0037 (7)	0.0031 (7)	0.0027 (7)
C17	0.0381 (12)	0.0765 (16)	0.112 (2)	0.0059 (10)	−0.0078 (12)	0.0371 (15)
C18	0.0805 (17)	0.0893 (18)	0.0625 (14)	−0.0134 (14)	−0.0233 (13)	−0.0233 (13)
C19	0.0412 (10)	0.0394 (9)	0.0440 (10)	0.0015 (8)	0.0007 (8)	0.0025 (8)
C20	0.0319 (8)	0.0391 (9)	0.0374 (9)	0.0023 (7)	−0.0010 (7)	0.0045 (7)
C21	0.0402 (10)	0.0481 (10)	0.0374 (9)	−0.0012 (8)	0.0049 (7)	0.0027 (8)
C22	0.0486 (11)	0.0456 (10)	0.0387 (9)	−0.0015 (8)	0.0012 (8)	−0.0048 (8)
C23	0.0354 (9)	0.0396 (9)	0.0455 (10)	−0.0014 (7)	−0.0028 (8)	0.0044 (8)
C24	0.0346 (9)	0.0463 (10)	0.0420 (9)	0.0024 (8)	0.0055 (7)	0.0066 (8)
C25	0.0383 (9)	0.0406 (9)	0.0386 (9)	0.0033 (7)	0.0031 (7)	−0.0004 (7)

### Geometric parameters (Å, °)

**Table:**

Mn1—O2	1.8821 (11)	C8—C9	1.507 (3)
Mn1—O1	1.8848 (12)	C8—H8A	0.9900
Mn1—N1	1.9774 (15)	C8—H8B	0.9900
Mn1—N2	1.9930 (14)	C9—H9A	0.9900
Mn1—O4	2.1164 (13)	C9—H9B	0.9900
Mn1—O3	2.3257 (12)	C10—C11	1.437 (3)
O1—C1	1.318 (2)	C10—H10	0.9500
O2—C16	1.316 (2)	C11—C16	1.407 (2)
O3—H1W	0.8400	C11—C12	1.415 (3)
O3—H2W	0.8401	C12—C13	1.359 (3)
O4—C19	1.253 (2)	C12—H12	0.9500
O5—C19	1.236 (2)	C13—C14	1.392 (3)
O6—C2	1.366 (3)	C13—H13	0.9500
O6—C17	1.435 (2)	C14—C15	1.372 (3)
O7—C15	1.366 (2)	C14—H14	0.9500
O7—C18	1.424 (2)	C15—C16	1.419 (2)
O8—C23	1.358 (2)	C17—H17A	0.9800
O8—H8	0.8400	C17—H17B	0.9800
N1—C7	1.274 (2)	C17—H17C	0.9800
N1—C8	1.474 (2)	C18—H18A	0.9800
N2—C10	1.278 (2)	C18—H18B	0.9800
N2—C9	1.470 (2)	C18—H18C	0.9800
C1—C6	1.411 (3)	C19—C20	1.509 (2)
C1—C2	1.416 (3)	C20—C25	1.386 (2)
C2—C3	1.383 (3)	C20—C21	1.394 (2)
C3—C4	1.393 (4)	C21—C22	1.379 (3)
C3—H3A	0.9500	C21—H21	0.9500
C4—C5	1.352 (4)	C22—C23	1.391 (3)
C4—H4	0.9500	C22—H22	0.9500
C5—C6	1.410 (3)	C23—C24	1.385 (3)
C5—H5	0.9500	C24—C25	1.387 (3)
C6—C7	1.428 (3)	C24—H24	0.9500
C7—H7	0.9500	C25—H25	0.9500
			
O2—Mn1—O1	94.17 (5)	C8—C9—H9A	110.3
O2—Mn1—N1	172.37 (6)	N2—C9—H9B	110.3
O1—Mn1—N1	91.90 (6)	C8—C9—H9B	110.3
O2—Mn1—N2	90.95 (6)	H9A—C9—H9B	108.5
O1—Mn1—N2	172.26 (6)	N2—C10—C11	125.20 (16)
N1—Mn1—N2	82.56 (6)	N2—C10—H10	117.4
O2—Mn1—O4	89.99 (5)	C11—C10—H10	117.4
O1—Mn1—O4	98.52 (6)	C16—C11—C12	119.61 (18)
N1—Mn1—O4	93.68 (6)	C16—C11—C10	122.03 (16)
N2—Mn1—O4	87.26 (6)	C12—C11—C10	118.25 (17)
O2—Mn1—O3	90.40 (5)	C13—C12—C11	120.9 (2)
O1—Mn1—O3	91.09 (5)	C13—C12—H12	119.6
N1—Mn1—O3	84.87 (6)	C11—C12—H12	119.6
N2—Mn1—O3	83.08 (5)	C12—C13—C14	120.26 (19)
O4—Mn1—O3	170.33 (5)	C12—C13—H13	119.9
C1—O1—Mn1	128.61 (12)	C14—C13—H13	119.9
C16—O2—Mn1	126.94 (10)	C15—C14—C13	120.29 (19)
Mn1—O3—H1W	109.5	C15—C14—H14	119.9
Mn1—O3—H2W	109.5	C13—C14—H14	119.9
H1W—O3—H2W	98.5	O7—C15—C14	125.68 (17)
C19—O4—Mn1	137.94 (13)	O7—C15—C16	113.16 (15)
C2—O6—C17	118.09 (18)	C14—C15—C16	121.16 (18)
C15—O7—C18	118.02 (17)	O2—C16—C11	124.73 (15)
C23—O8—H8	109.5	O2—C16—C15	117.41 (15)
C7—N1—C8	120.99 (16)	C11—C16—C15	117.83 (16)
C7—N1—Mn1	126.33 (14)	O6—C17—H17A	109.5
C8—N1—Mn1	112.66 (12)	O6—C17—H17B	109.5
C10—N2—C9	122.20 (16)	H17A—C17—H17B	109.5
C10—N2—Mn1	125.41 (12)	O6—C17—H17C	109.5
C9—N2—Mn1	112.39 (12)	H17A—C17—H17C	109.5
O1—C1—C6	124.27 (17)	H17B—C17—H17C	109.5
O1—C1—C2	117.72 (18)	O7—C18—H18A	109.5
C6—C1—C2	118.01 (18)	O7—C18—H18B	109.5
O6—C2—C3	125.8 (2)	H18A—C18—H18B	109.5
O6—C2—C1	113.84 (17)	O7—C18—H18C	109.5
C3—C2—C1	120.4 (2)	H18A—C18—H18C	109.5
C2—C3—C4	120.8 (2)	H18B—C18—H18C	109.5
C2—C3—H3A	119.6	O5—C19—O4	124.60 (18)
C4—C3—H3A	119.6	O5—C19—C20	120.33 (16)
C5—C4—C3	119.9 (2)	O4—C19—C20	115.06 (16)
C5—C4—H4	120.0	C25—C20—C21	118.07 (16)
C3—C4—H4	120.0	C25—C20—C19	122.08 (16)
C4—C5—C6	121.3 (2)	C21—C20—C19	119.86 (15)
C4—C5—H5	119.4	C22—C21—C20	121.03 (16)
C6—C5—H5	119.4	C22—C21—H21	119.5
C5—C6—C1	119.7 (2)	C20—C21—H21	119.5
C5—C6—C7	117.6 (2)	C21—C22—C23	120.13 (17)
C1—C6—C7	122.67 (17)	C21—C22—H22	119.9
N1—C7—C6	125.66 (18)	C23—C22—H22	119.9
N1—C7—H7	117.2	O8—C23—C24	123.75 (16)
C6—C7—H7	117.2	O8—C23—C22	116.63 (17)
N1—C8—C9	107.53 (16)	C24—C23—C22	119.62 (16)
N1—C8—H8A	110.2	C23—C24—C25	119.58 (16)
C9—C8—H8A	110.2	C23—C24—H24	120.2
N1—C8—H8B	110.2	C25—C24—H24	120.2
C9—C8—H8B	110.2	C20—C25—C24	121.55 (16)
H8A—C8—H8B	108.5	C20—C25—H25	119.2
N2—C9—C8	107.24 (16)	C24—C25—H25	119.2
N2—C9—H9A	110.3		
			
O2—Mn1—O1—C1	167.31 (14)	Mn1—N1—C7—C6	−1.8 (3)
N1—Mn1—O1—C1	−8.07 (15)	C5—C6—C7—N1	177.95 (19)
O4—Mn1—O1—C1	−102.08 (15)	C1—C6—C7—N1	−3.2 (3)
O3—Mn1—O1—C1	76.83 (14)	C7—N1—C8—C9	145.79 (18)
O1—Mn1—O2—C16	162.00 (13)	Mn1—N1—C8—C9	−35.67 (19)
N2—Mn1—O2—C16	−23.80 (14)	C10—N2—C9—C8	143.81 (18)
O4—Mn1—O2—C16	63.46 (14)	Mn1—N2—C9—C8	−35.78 (19)
O3—Mn1—O2—C16	−106.88 (13)	N1—C8—C9—N2	45.1 (2)
O2—Mn1—O4—C19	140.4 (2)	C9—N2—C10—C11	174.25 (17)
O1—Mn1—O4—C19	46.2 (2)	Mn1—N2—C10—C11	−6.2 (3)
N1—Mn1—O4—C19	−46.3 (2)	N2—C10—C11—C16	−7.3 (3)
N2—Mn1—O4—C19	−128.6 (2)	N2—C10—C11—C12	176.57 (18)
O1—Mn1—N1—C7	6.06 (17)	C16—C11—C12—C13	0.2 (3)
N2—Mn1—N1—C7	−168.52 (17)	C10—C11—C12—C13	176.4 (2)
O4—Mn1—N1—C7	104.73 (16)	C11—C12—C13—C14	0.0 (3)
O3—Mn1—N1—C7	−84.86 (16)	C12—C13—C14—C15	0.2 (3)
O1—Mn1—N1—C8	−172.39 (13)	C18—O7—C15—C14	−3.6 (3)
N2—Mn1—N1—C8	13.03 (13)	C18—O7—C15—C16	177.24 (18)
O4—Mn1—N1—C8	−73.72 (13)	C13—C14—C15—O7	−179.66 (19)
O3—Mn1—N1—C8	96.69 (13)	C13—C14—C15—C16	−0.6 (3)
O2—Mn1—N2—C10	17.80 (15)	Mn1—O2—C16—C11	18.7 (2)
N1—Mn1—N2—C10	−166.21 (16)	Mn1—O2—C16—C15	−163.44 (12)
O4—Mn1—N2—C10	−72.14 (15)	C12—C11—C16—O2	177.39 (17)
O3—Mn1—N2—C10	108.09 (15)	C10—C11—C16—O2	1.3 (3)
O2—Mn1—N2—C9	−162.63 (13)	C12—C11—C16—C15	−0.5 (3)
N1—Mn1—N2—C9	13.36 (13)	C10—C11—C16—C15	−176.59 (16)
O4—Mn1—N2—C9	107.43 (13)	O7—C15—C16—O2	1.9 (2)
O3—Mn1—N2—C9	−72.34 (13)	C14—C15—C16—O2	−177.34 (17)
Mn1—O1—C1—C6	5.9 (3)	O7—C15—C16—C11	179.91 (15)
Mn1—O1—C1—C2	−174.29 (12)	C14—C15—C16—C11	0.7 (3)
C17—O6—C2—C3	7.6 (3)	Mn1—O4—C19—O5	21.3 (3)
C17—O6—C2—C1	−172.48 (17)	Mn1—O4—C19—C20	−159.68 (14)
O1—C1—C2—O6	0.6 (2)	O5—C19—C20—C25	14.1 (3)
C6—C1—C2—O6	−179.62 (16)	O4—C19—C20—C25	−164.99 (17)
O1—C1—C2—C3	−179.53 (17)	O5—C19—C20—C21	−165.88 (18)
C6—C1—C2—C3	0.3 (3)	O4—C19—C20—C21	15.1 (2)
O6—C2—C3—C4	178.98 (19)	C25—C20—C21—C22	−0.8 (3)
C1—C2—C3—C4	−0.9 (3)	C19—C20—C21—C22	179.17 (17)
C2—C3—C4—C5	1.0 (3)	C20—C21—C22—C23	1.5 (3)
C3—C4—C5—C6	−0.4 (3)	C21—C22—C23—O8	179.52 (17)
C4—C5—C6—C1	−0.2 (3)	C21—C22—C23—C24	−0.9 (3)
C4—C5—C6—C7	178.7 (2)	O8—C23—C24—C25	179.18 (16)
O1—C1—C6—C5	−179.94 (17)	C22—C23—C24—C25	−0.4 (3)
C2—C1—C6—C5	0.2 (3)	C21—C20—C25—C24	−0.5 (3)
O1—C1—C6—C7	1.3 (3)	C19—C20—C25—C24	179.53 (15)
C2—C1—C6—C7	−178.54 (17)	C23—C24—C25—C20	1.1 (3)
C8—N1—C7—C6	176.51 (19)		

### Hydrogen-bond geometry (Å, °)

**Table:**

*D*—H···*A*	*D*—H	H···*A*	*D*···*A*	*D*—H···*A*
O8—H8···O5^i^	0.84	1.79	2.599 (2)	161
O3—H2W···O7^ii^	0.84	2.32	3.0025 (19)	139
O3—H2W···O2^ii^	0.84	2.11	2.8711 (17)	150
O3—H1W···O6^ii^	0.84	2.29	3.000 (2)	142
O3—H1W···O1^ii^	0.84	2.21	2.9475 (18)	147

Symmetry codes: (i) −*x*+1, *y*+1/2, −*z*+1/2; (ii) −*x*, −*y*+1, −*z*+1.
